# Thresholds for blood transfusion in extremely preterm infants: A review of the latest evidence from two large clinical trials

**DOI:** 10.3389/fped.2022.957585

**Published:** 2022-09-20

**Authors:** Michael P. Meyer, Kristin L. O'Connor, Jill H. Meyer

**Affiliations:** ^1^Neonatal Unit, KidzFirst, Middlemore Hospital, Auckland, New Zealand; ^2^Department of Paediatrics Child and Youth Health, University of Auckland, Auckland, New Zealand; ^3^Department of Biomedicine and Medical Diagnostics, Auckland University of Technology, Auckland, New Zealand

**Keywords:** preterm, blood transfusion, review, hemoglobin thresholds, latest evidence

## Abstract

There are two recently completed large randomized clinical trials of blood transfusions in the preterm infants most at risk of requiring them. Liberal and restrictive strategies were compared with composite primary outcome measures of death and neurodevelopmental impairment. Infants managed under restrictive guidelines fared no worse in regard to mortality and neurodevelopment in early life. The studies had remarkably similar demographics and used similar transfusion guidelines. In both, there were fewer transfusions in the restrictive arm. Nevertheless, there were large differences between the studies in regard to transfusion exposure with almost 3 times the number of transfusions per participant in the transfusion of prematures (TOP) study. Associated with this, there were differences between the studies in various outcomes. For example, the combined primary outcome of death or neurodevelopmental impairment was more likely to occur in the TOP study and the mortality rate itself was considerably higher. Whilst the reasons for these differences are likely multifactorial, it does raise the question as to whether they could be related to the transfusions themselves? Clearly, every effort should be made to reduce exposure to transfusions and this was more successful in the Effects of Transfusion Thresholds on Neurocognitive Outcomes (ETTNO) study. In this review, we look at factors which may explain these transfusion differences and the differences in outcomes, in particular neurodevelopment at age 2 years. In choosing which guidelines to follow, centers using liberal guidelines should be encouraged to adopt more restrictive ones. However, should centers with more restrictive guidelines change to ones similar to those in the studies? The evidence for this is less compelling, particularly given the wide range of transfusion exposure between studies. Individual centers already using restrictive guidelines should assess the validity of the findings in light of their own transfusion experience. In addition, it should be remembered that the study guidelines were pragmatic and acceptable to a large number of centers. The major focus in these guidelines was on hemoglobin levels which do not necessarily reflect tissue oxygenation. Other factors such as the level of erythropoiesis should also be taken into account before deciding whether to transfuse.

## Introduction

Blood transfusions have been a common therapeutic practice worldwide for many decades. In recent times, evidence is pointing away from the use of transfusion in both adult and pediatric intensive care with a reduction in mortality in critically ill patients (1, 2). In contrast, however, it appears that there is no difference between outcomes from the administration of liberal vs. restrictive red cell transfusion in less critical situations (3). In fact, a restrictive red cell transfusion strategy may be at least as safe as a liberal strategy and may even reduce patient mortality in specific patient subpopulations (it should be noted that the definitions of “liberal” and “restrictive” practices are, to some extent, arbitrary and based on hemoglobin thresholds with higher thresholds e.g., 90–100 g/L for “liberal” and lower ones e.g., 70–80 g/L for “restrictive”) (3). Exactly why blood transfusions appear more harmful in the critically ill patient is uncertain, but the harmful effect maybe from pro inflammatory and immunosuppressive effects of transfusion (TRIM or Transfusion Related Immuno Modulation) (4).

In preterm infants, while blood transfusion can be life-saving in a scenario of acute blood loss, more usually the context for transfusion includes an asymptomatic preterm infant with low hemoglobin, known as Anemia of Prematurity. This has a multi-factorial etiology where normochromic normocytic erythrocytes are associated with an inappropriately low reticulocyte count and erythropoietin levels. Red cell survival is usually also reduced. In addition, there is the overlay of blood sampling losses in a neonatal intensive care unit. Often in such premature infants, the anemia is further compounded by iron or other nutritional deficiency and/or infection, all in the setting of rapid growth demands (5).

Whilst transfusions are common in preterm infants, there is considerable variability with one study reporting that over 90% of infants <1,000 g received at least one transfusion during the neonatal admission whilst another reported a rate of 40%. Various attempts to provide clinical guidelines have been made, but these are by no means sharply defined, and several outstanding concerns persist, including those regarding longer-term sequelae (6).

The source of such major variation in transfusion practices has in part been due to a lack of specific markers of tissue oxygenation. Most decisions to transfuse center on the level of hemoglobin, but this is only one factor. What is really needed is an overall assessment of tissue oxygen demand, delivery and consumption. Measurement of near-infra-red spectroscopy holds promise in this regard (7).

In addition, until recently, there has also been a lack of high quality randomized controlled trials (RCTs) to inform transfusion practice in neonatology. However, this has recently changed and there are now two high quality, adequately powered RCTs to guide management The two studies, the Transfusion of Prematures (TOP) trial (8) and effects of liberal vs. restrictive transfusion thresholds on survival and neurocognitive outcomes in extremely low birth weight infants (ETTNO) (9), are likely to inform practice about 2 years neurodevelopmental (ND)/ mortality outcomes and blood transfusion thresholds for some time to come.

## Trial summaries and primary outcomes

The ETTNO trial randomized 1,013 neonates, all less than 72 h of age, across 36 NICUs in Europe (32 NICUs in Germany), with birth weights between 400 and 999 g, <30 weeks gestational age and carried out long term follow up at 24 months corrected (± 1 month). Primary outcome was death or disability at this time. The TOP trial included 1,692 neonates with birth weights <1,000 g and gestational ages between 22 weeks 0 days and 28 weeks 6 days. Neonates were randomized within 48 h after delivery; all study centers were in the USA (Table 1). Primary outcomes were a composite of death or neurodevelopmental impairment at 22–26 months corrected age. Both studies had similar exclusion criteria and the definitions of primary outcomes differed partly in the classification of cerebral palsy, TOP using the GMFCS (Gross Motor Function Classification System) and ETTNO using the Surveillance of CP in Europe network definitions. In addition, TOP used a Bayley Scale of Infant Development (BSID) III assessment and ETTNO BSID-II.

**Table 1:** Comparison between ETTNO and TOP trials and between high and low threshold groups at randomization and 36 weeks post menstrual age (PMA).

	**ETTNO**		**TOP**	
	**High (*n* = 492)**	**Low (*n* = 521)**	**High (*n* = 845)**	**Low (*n* = 847)**
GA (weeks)	26.1 (IQR 24.9-27.6)	26.4 (IQR 25-27.6)	25.9 (SD 1.5)	25.9 (SD 1.5)
BW (g) mean ± SD	753 ± 164	750 ± 163	755.2 ± 152.7	757.2 ± 147.7
<750 g (%)	51	49	46	46
750–1,000 g (%)	49	51	54	54
**Growth parameters at 36wks PMA (mean SD)**				
Weight (g)	2113 ± 356	2068 ± 361	2106 ± 398	2098 ±367
HC (cm)	30.6 ±1.8	30.5 ±1.8	30.4 ±1.8	30.4 ± 1.7
Length (cm)	42.8 ± 2.7	42.5 ± 2.7	42.1 ± 2.9	42.0 ± 2.7
Any Antenatal steroids given (%)	89	88	91	89
Delayed cord clamping or milking (%)	63	61	27	24
Hb at randomization g/dL (mean ± SD)	15.8 ± 2.4	16.1 ± 2.4	13.8 ± 2.6	13.7 ± 2.6
Hb at 36 wks PMA or discharge g/dL (mean ± SD)	12.4 ± 2.3	11.2 ± 1.2	11.5 ± 1.6	10.2 ± 1.4

GA, gestational age; BW, birth weight; Hb, hemoglobin.

The composite primary outcomes for both trials showed no difference between the high and lower threshold groups (Table 2). Separation between groups regarding hemoglobin levels or hematocrit was greater in TOP (19 g/L in TOP and, converted from hematocrit, 9 g/L in ETTNO). Transfusions given outside of protocol amounted to 23% of all transfusions in the TOP restrictive group and similarly 19% of infants in the ETTNO study restrictive group had at least one transfusion outside of protocol. However, a considerable number of these were thought to be clinically justified. Blinding of assignment allocation was not possible but outcome assessors were masked as to treatment group. Overall, the findings of these two large randomized studies clearly showed no benefit using liberal transfusion thresholds within the hemoglobin ranges studied. These findings are summarized in Table 2 and have recently been reviewed (10).

**Table 2:** Comparison of primary and secondary outcomes in restrictive and liberal transfusion groups in ETTNO and TOP studies.

	**ETTNO restrictive threshold (*n* = 478)**	**TOP restrictive threshold (*n* = 847)**	**ETTNO liberal threshold (*n* = 450)**	**TOP liberal threshold (*n* = 845)**
**Primary outcome (%)**				
Composite of death or neurodevelopmental impairment	42.9	49.8	44.4	50.1
Components of primary outcome				
Death	9	15	8.3	16.2
Neurodevelopmental impairment				
Cognitive delay	34.4	37.9	37.6	38.7
Moderate or severe cerebral palsy	5.6	7.6	4.3	6.8
severe vision impairment	2.7	0.8	2.4	0.7
Severe hearing impairment	1.4	3.5	1.0	2.0
**Secondary outcomes (%)**				
BPD	26	56.3	28.4	59
Necrotizing enterocolitis Bells stage ≥2	6.2	10.5	5.3	10
Retinopathy of prematurity stage 3 or surgical intervention	13	17.2	15.9	19.7
Sepsis	23.8	25.6	23.0	25.5
IVH grade 3–4	6.7	17.9	8.1	17.1
Cystic PVL or ventriculomegaly	5.8		4.7	

IVH, intraventricular hemorrhage; CPVL, cystic periventricular leukomalacia.

## Blood transfusion, neurological and neonatal outcomes

In spite of the similarity of the enrolment criteria and transfusion guidelines (as shown in Table 3 with the ETTNO trial thresholds converted to Hb g/dL from hematocrit for ease of comparison), there were differences between the transfusion rates and several neonatal outcomes in the two studies. Of 847 TOP trial infants in the restrictive group, 4,055 transfusions were given (4.8/participant) compared to 904 transfusions to 521 ETTNO trial infants (1.7/participant). In addition, a smaller percentage of TOP infants never received a transfusion (Table 4). There were substantial differences between studies in the performance of DCC or cord milking with over 60% reported in the ETTNO study and <30% in the TOP study. In the ETTNO study 25% of infants received a transfusion prior to randomization although it is not stated what proportion of these infants received DCC. It is noteworthy that 5% or fewer of the TOP participants were transfused before study commencement. Randomization in TOP occurred prior to 48 h and ETTNO by 72 h so this may partly explain the difference but it is unclear why there was such a large difference in early transfusion.

**Table 3:** Direct comparison of study guideline hemoglobin thresholds for ETTNO and TOP trials (g/dL).

**Postnatal age**	ETTNO*				**TOP**			
	**Liberal**		**Restricted**		**Liberal**		**Restricted**	
	**Critical**	**Non-critical**	**Critical**	**Non-critical**	**Respiratory support**	**No respiratory support**	**Respiratory support**	**No respiratory support**
Randomization−7 days	13.7	11.6	11.3	9.4	13	12	11	10
8–14 days	12.2	10.3	10	8.1	12.5	11	10	8.5
14–21days	12.2	10.3	10	8.1	11	10	8.5	7
>21 days	11.3	9.4	9	6.9	11	10	8.5	7

*The ETTNO values were converted from hematocrit to hemoglobin using a conversion of 0.3.

**Table 4:** Transfusion outcomes comparing ETTNO and TOP studies in the high and low threshold groups. Results shown as median (IQR) or mean (SD).

	**ETTNO (total transfusions** = **2,162)**		**TOP (total transfusions** = **9,679)**	
	**High (*n* = 1,258)**	**Low (*n* = 904)**	**High (*n* = 5,624)**	**Low (*n* = 4,055)**
Never transfused (%)	21	40	2.9	11.9
Patients transfused prior to inclusion in study (%)	25	24	5	4
Number of transfusions per infant	2 (1–4)	1 (0–3)	6.2 (4.3)	4.4 (4.0)
Total number of transfusions to 36 weeks PMA	1,258	904	5,624	4,055
Patients who received at least one RBC not on protocol (%)	10	19		
Non-protocol compliant transfusions (%)			5	23
Unjustified, non-protocol transfusions (violations) (%)	5	15	0.8	7.4
Clinically justified, non-protocol transfusions (%)	6	21	4	16
Number transfusion missed despite Hb trigger met	(no. of pt %)		(no. of transfusions %)	
	13	1	3.3	1

Hb, hemoglobin; Pt, participants.

Comparing the studies, there were differences in rates of death, bronchopulmonary dysplasia (BPD), necrotizing enterocolitis (NEC), 2 year neurodevelopmental (ND) outcome and ND impairment (NDI). The studies were conducted in different populations with different clinical practices. Nevertheless, because the studies had such similar enrollment criteria it is useful to compare outcomes to identify best practice and highlight areas for future research.

## Factors impacting differences in transfusion rates

There are various determinants of hemoglobin level (and hence transfusion) including:

Deferred cord clamping (DCC).

Phlebotomy losses.

Nutrition and iron supplements.

History of previous transfusions and suppression of erythropoietin levels.

## Deferred cord clamping (DCC)

As noted above, there were substantial differences between studies in the preformance of DCC or cord milking. A substantial body of evidence indicates a reduction in blood transfusions given to preterm infants (including the <1,000 g subgroup) after delayed cord clamping. One recent systematic review noted a 27% lower relative risk (RR) for any transfusion (RR 0.83; 95% CI 0.77–0.90) as well as a reduced mean number of transfusions for infants <34 weeks (mean difference −0.63; 95% CI −1.08 to −0.17) (11). In another systematic review infants <1,000 g had a RR for transfusion of 0.91 (CI 0.85–0.97) following DCC (12). Reasons for this include a significantly higher hematocrit and hemoglobin in the 1st 24 h (2.6% and 1.2 g respectively) (11). The hemoglobin level at randomization was 2–3 g/L higher in the ETTNO study likely reflecting differences in cord management. Other studies have noted an association between higher birth hematocrit and hemoglobin levels and reduction in neonatal transfusion (13, 14). Reduced need for inotrope support after birth has also been noted following DCC (11) and this was associated with a reduction in early transfusion in the UK cord trial (15). Carrying out cord clamping only after a period of established respiration has also been proposed as a mechanism of stabilizing transition after birth and this could be very important in extremely preterm infants (16).

It has been noted that even when DCC is intended, it cannot be achieved in 30–40% of preterm infants for a variety of maternal or infant reasons (17).

## Phlebotomy losses

These were not reported in the studies, although there is a plan to report these in the ETTNO study.

A large number of studies have noted associations between blood sampling volumes and subsequent transfusion. Using regression models, a recent study in VLBW infants noted a significant association between transfusion, sampling losses and birth hematocrit (13). Whilst every attempt should be made to minimize iatrogenic blood losses there are no specific targets and comparisons between units are difficult partly because of different reporting of losses (e.g., total or ml per kg over a specified period). For example, in infants <1,000 g studied in one USA center, the median total losses were 83 ml in those whose length of stay was over 10 weeks compared to another USA study which reported losses of 10–25 ml/week. (14, 18). This is in comparison to an earlier placebo-controlled study of erythropoietin use where median total losses over the hospital stay in infants <30 weeks was 20 ml (19). With such vast differences in sampling losses in these previous studies, it is not surprising that reported transfusion rates were also very different. It is possible that there were differences in sampling losses in the TOP and ETTNO trials, which might be another explanation for the transfusion differences between the two studies.

Quality improvement projects (for example using a bundle-of-care approach) have resulted in reduction in iatrogenic blood loss and one such study noted a significant reduction in early blood losses in ELBW infants (20).

Apart from a rationalized approach with a reduction in unnecessary tests, the method of laboratory testing is also very important. For example, in-line, sampling devices measured blood gases, hematocrit, and electrolytes using very small volumes of blood compared to standard point-of-care testing. Other micro-methods have included dried blood testing and volumetric absorption micro sampling (VAMS). With these techniques only 10–30 microliters may be required compared to standard amounts of up to 500 microliters (21). Point of care testing has also been associated with reductions in phlebotomy losses and transfusion (22–24).

## Guidelines

As shown in Table 3, the guidelines followed in the two studies were similar, taking the age and illness severity into account as well as hemoglobin levels. In the TOP study, illness severity was based on respiratory support. ETTNO was similar but with slightly broader criteria and included NEC. In spite of the similarity in the guidelines, ~20% of transfusions in the restrictive group in both studies were non-protocol compliant. Overall, this suggests many clinicians might not have been comfortable with lower thresholds and highlights the difficulty of ensuring compliance with guidelines, even under the scrutiny of a clinical trial. In addition, as more transfusions were given in the restrictive groups this would reduce study power to detect a true difference between the liberal and restrictive arms of the studies.

Nevertheless, it is important to have guidelines as there are studies indicating a reduction of transfusions when guidelines are applied (25). There are currently wide differences in guideline recommendations from different countries and neonatal units. These have been reviewed in two recent publications (26, 27). Similarly, in the Preterm Erythropoietin Neuroprotection Trial (PENUT) study comparing erythropoietin with placebo for preterm neurodevelopment, it was noted that there was substantial variability in transfusion practice by site and only 74% of participating sites used formal transfusion guidelines (28).

The guidelines used in the 2 studies have the advantage that they have 2 year outcomes associated with them and the results strongly indicate that restrictive guidelines can safely be used. This has potential to influence transfusion policy going forward so that centers using more liberal thresholds can confidently change practice. However, it should be noted that the study guidelines were largely pragmatic, had to be agreed upon by multiple centers and clearly did not cover all scenarios. Physiologically, attempting to define a low hemoglobin threshold that is generally appropriate for the preterm population is difficult because tissue oxygenation does not depend on hemoglobin level alone. It would seem important to consider factors other than those in the guidelines e.g., history of previous transfusion and reticulocyte counts as an indication of marrow responsiveness. The studies did not allow use of erythrocyte stimulating agents (ESA) such as erythropoietin. There seems little disadvantage to using ESAs, especially with reduced concerns for an effect on retinopathy of prematurity and a recent systematic review showing improvement in neurodevelopment (29).

## Ventilation strategy

The type of respiratory support e.g., CPAP or mechanical ventilation as well as the presence of an umbilical arterial catheter have been shown to influence phlebotomy losses, which may, in turn, alter transfusion requirements (30, 31). Whilst it is plausible that the respiratory support strategy may influence transfusion rates, evidence from RCTs is lacking. Two large RCTs comparing respiratory support strategies did not report transfusion outcomes (32, 33). In the ETTNO study, 60% of participants were managed with CPAP or a non-invasive strategy, but this information was not provided in the TOP study so comparisons cannot be drawn but it may be worth further study in future.

## Nutrition

Although protein intakes were not specifically reported in either study, higher intakes have been associated with an increase in hemoglobin and erythropoiesis (34, 35). In the latter small study, intakes above 3.5 g/kg/d did not result in further increases in reticulocyte count in Very Low Birth Weight Infants over a 6 week period.

Oral iron supplementation has been linked to improved hemoglobin levels and reduced anemia in preterm and low birth weight infants in the first months of life (36). However, several studies have reported no association between transfusion requirements in preterm infants and iron supplementation, and the anemia of prematurity is not primarily one of iron deficiency (37, 38).

Although neither study reported these nutritional aspects in detail, it is unlikely there was a significant impact on transfusion outcomes, although it would seem important to have more information to assess any impact on longer term ND. An analysis of iron supplementation in the PENUT study indicated a positive association between cumulative iron dosage at 60 days and BSID-III score at 2 years of age (39).

## Possible effects of transfusion differences on outcomes

### Neurodevelopmental outcomes

As noted above, there were no differences between the liberal and restrictive arms of either study in terms of neurodevelopment (ND). Cognitive delay was 10% higher in TOP than ETTNO and moderate to severe cerebral palsy rates were 35% higher in TOP. However, direct comparison of the 2 year ND between the studies (inter-study) is compounded by different Bayley assessment templates (II vs. III) and different cerebral palsy definitions. Having standardized assessments would make the data more comparable and it will be important to continue the follow up for longer term outcomes, e.g., at school age. The observed differences raise the question as to whether transfusion exposure is related to ND.

In the Preterm Erythropoietin Neuroprotection Trial (28), the effect of transfusion on ND outcome at 22–24 months of age was investigated as a secondary outcome in preterm infants <28 weeks gestation (28). Comparing outcomes of infants who did not receive any transfusions with those who received one or more (and after adjusting for covariates), small but significant decreases in mean cognitive, mean motor and mean language scores were noted. Associations for severe and moderate NDI were also examined (severe CP, BSID-III cognitive <70, BSID-III motor <70 and moderate-to-severe NDI, moderate-to-severe CP, BSID-III cognitive <85, BSID-III motor <85). Worse outcomes were significantly and strikingly associated with the number and volume of transfusions and number of donors. Limitations of this study, however, include its observational nature and the difficulty of separating association of transfusion with severity of illness.

Follow up of preterm infants (500–1,250 g) at 18–22 months who were involved in a randomized study investigating use of ESAs indicated a negative correlation between BSID-III scores and volume of transfusion (*r* = 0.26; *p* = 0.02) (40).

In another recent (observational) study of preterm infants <34 weeks gestation, BSID-III scores for cognitive and motor domains at 18-36 months were negatively associated with receiving a blood transfusion. The number of transfusions was associated with an increase in severe ND impairment (adjusted-odds ratio 1.09; 95% confidence interval 1.03–1.15) (41).

However, against this possible link is the fact that the intra-study differences in transfusion rates per patient between high and low threshold group in both the ETTNO (2 vs. 1) and TOP studies (6 vs. 4), although significant, did not appear to affect ND outcomes. The PINTOS study (42) was an 18–21 month follow up of the PINT study (43), which compared higher and lower transfusion thresholds in extremely preterm infants. The PINTOS study found a small but significant increase of the Bayley II MDI of 4 points in the higher threshold group.

Longer term outcomes have also been reported. A study reporting follow up at school age of a randomized trial comparing a liberal and restrictive transfusion strategy noted that preterm infants in the liberal transfusion arm had worse outcomes in regards to verbal fluency, visual memory and reading than those in the restrictive group (44). A subgroup of 26 infants in the liberal transfusion group underwent further testing. It was shown that poor verbal fluency in females was associated with reduced temporal lobe white matter on MRI (45). Lower erythropoietin levels following transfusion have been proposed as a potential mechanism for these findings.

Overall there remains uncertainty about the effect of transfusion on 2 year ND outcome. In addition, longer term outcomes may be different to the 2 year follow up data. Apart from the possible effect of confounding by illness severity, a potential explanation is that of competing outcomes. Although transfusion may have a negative effect on ND outcome, raising hemoglobin levels by transfusion may overcome adverse effects from cerebral hypoxia. A potential way forward to provide insight into this is the use of Near Infrared Spectroscopy (NIRS), as further discussed below.

### Necrotizing enterocolitis

There were marked differences in the studies in relation to necrotizing enterocolitis (NEC) stage 2 or more. These differences are no doubt multifactorial, including use of probiotic prophylaxis in ETTNO patients. The larger number of transfusions in TOP could be linked to an increase in NEC rates although a comparison of the liberal and restrictive groups in TOP did not show an increase in NEC rates with the increased transfusion exposure. Many observational studies have reported a temporal association between transfusion and NEC but this has not been a consistent finding (26). Analysis of the 72 h period following transfusion in the TOP study found no temporal association between NEC and transfusion (46). Another potential mechanism is that a low hemoglobin level could lead to gut hypoxia with a reperfusion injury and NEC following transfusion (47). The two transfusion studies could not shed further light on these mechanisms but the inter-study differences are notable.

### Mortality

The mortality rate in the ETTNO study was considerably lower than in TOP. The rate in the former study was notably lower than that reported from another study also carried out in the same neonatal network (48). The authors of ETTNO speculate that parents whose infants were unstable may have declined to participate, although the same limitation probably applied to the TOP study. It might be expected that any effect of transfusion on mortality would have shown at least a trend toward lower mortality in the restrictive group, but this was not the case. Probiotics, as used in the ETTNO study, have also been associated with a reduction in mortality.

### Other outcomes (ROP and BPD)

As shown in Table 2 rates of stage 3 or more ROP and BPD were also different between studies. Although the definition of the latter was slightly different between studies, it is unlikely this would account for rates that were more than double in the TOP study. As previously noted above in relation to some of the other neonatal outcomes, it is uncertain if the different transfusion rates had an influence on these outcomes. Nevertheless, there are studies that have noted an association between transfusion rates and ROP, but this has not been a consistent finding (26) and between transfusion and BPD (49).

## The way forward—An individualized approach to transfusion?

Prior to the two studies, published transfusion guidelines were often more liberal and targeted higher hemoglobin levels (26, 27). However, in the 2 studies, there was no evidence of worse neonatal outcomes in preterm infants with a restrictive approach using the lower hemoglobin thresholds.

For centers using a more liberal approach than those in the TOP and ETTNO guidelines, the more restrictive thresholds are warranted. What about centers using a more restrictive approach (than the study thresholds)? Should they adopt the study guidelines (and become more liberal)? The studies are not able to answer this question. Whilst being more restrictive could potentially avoid any adverse neurodevelopment or other neonatal outcomes that are transfusion associated, there could be competing outcomes where lower hemoglobin levels cause tissue hypoxia. Ongoing surveillance and benchmarking of neonatal and neurodevelopmental outcomes will be important to show absence of harm in such centers.

It is worth pointing out that efforts to improve hemoglobin levels, such as those mentioned in this review will lead to a reduced need to consider transfusion at all. Fewer transfusions will cause less suppression of endogenous erythropoietin levels.

The study guidelines themselves provide hemoglobin thresholds but other factors not considered in the study guidelines that could influence a transfusion decision include previous history of transfusion, reticulocyte counts, expected phlebotomy losses, nutrition, other illnesses and their severity, and the use of ESAs. In the future, use of NIRS may help determine tissue hypoxia in real time.

Using NIRS, oxygen consumption can be measured as the fractional total oxygen extraction (FTOE) which is the ratio of consumption to supply. Although NIRS is often measured in the context of brain oxygenation, it can be used for other tissues as well, such as peripheral and gut perfusion. Increasing anemia has been associated with decreasing cerebral oxygen saturation or a compensatory increase in cerebral oxygen extraction (cFTOE) in several small observational studies, as noted in a recent review (7). Increasing cFTOE could potentially be a physiological marker of increasing anemia. In an observational longitudinal study, a modest correlation between cFTOE and hemoglobin was detected (*r* = −0.42). A threshold of cFTOE of greater than 0.4 (2SDs below the mean using normative data) was proposed as a cut-off. Above this, the risk of NDI may be increased (50). As preterm infants have a limited ability to autoregulate cerebral blood flow, it is conceivable that intercurrent illness or other events may affect autoregulation and, in the presence of anemia, could be associated with cerebral hypoxia. Although a promising tool, defining hemoglobin thresholds by this technique appears to be some way off but is being carried out on a subset of TOP study patients. Nevertheless, one of the attractive features is that it provides direct insight into tissue oxygenation for a given hemoglobin level in an individualized way.

Overall, the two studies will help reduce transfusion exposure but critical appraisal of how to improve hemoglobin levels and expand the transfusion guidelines to cover more clinical scenarios is required. It should be remembered that the study populations, whilst apparently representative, are influenced by the need for parents to provide informed consent and this is likely to affect results. In addition, participants were enrolled in the first few days after birth, so that extrapolation to the early period after birth is not possible.

We suggest careful consideration of these factors in putting these important studies into clinical practice.

## Author contributions

MM developed the idea for the review and wrote the initial draft of the article. KO'C and JM contributed to writing and editing the article. All authors contributed to the article and approved the submitted version.

## Conflict of interest

The authors declare that the research was conducted in the absence of any commercial or financial relationships that could be construed as a potential conflict of interest.

## Publisher's note

All claims expressed in this article are solely those of the authors and do not necessarily represent those of their affiliated organizations, or those of the publisher, the editors and the reviewers. Any product that may be evaluated in this article, or claim that may be made by its manufacturer, is not guaranteed or endorsed by the publisher.
